# Feasibility of Endoscopic Treatment of Middle Ear Myoclonus: A Cadaveric Study

**DOI:** 10.1155/2014/175268

**Published:** 2014-03-10

**Authors:** Natasha Pollak, Roya Azadarmaki, Sidrah Ahmad

**Affiliations:** ^1^Department of Otolaryngology-Head & Neck Surgery, Temple University School of Medicine, Philadelphia, PA 19140, USA; ^2^Metropolitan NeuroEar Group, Rockville, MD 20852, USA

## Abstract

Stapedius and tensor tympani tenotomy is a relatively simple surgical procedure commonly performed to control pulsatile tinnitus due to middle ear myoclonus and for several other indications. We designed a cadaveric study to assess the feasibility of an entirely endoscopic approach to stapedius and tensor tympani tenotomy. We performed this endoscopic ear surgery in 10 cadaveric temporal bones and summarized our experience. Endoscopic stapedius and tensor tympani section is a new, minimally invasive treatment option for middle ear myoclonus that should be considered as the first line surgical approach in patients who fail medical therapy. The use of an endoscopic approach allows for easier access and vastly superior visualization of the relevant anatomy, which in turn allows the surgeon to minimize tissue dissection. The entire operation, including raising the tympanomeatal flap and tendon section, can be safely completed under visualization with a rigid endoscope.

## 1. Introduction

Middle ear myoclonus is an infrequent but well-known cause of pulsatile tinnitus. It was first described by Adam Politzer in the late 19th century and is still not a clearly understood clinical entity. Occasionally, a causative lesion can be identified on magnetic resonance imaging (MRI) in the Guillain-Mollaret triangle in the brainstem and cerebellum, also known as the myoclonic triangle; however, most cases are idiopathic. The quality of pulsatile tinnitus associated with middle ear myoclonus is variable and described by patients most frequently as crackling, but also clicking, tapping, thumping, pulsations, fluttering moth, machinery rumble, and whooshing sounds. When myoclonus is slower, individual clicks can be discerned, once the pace of muscle contraction is faster, the sound blends into one continuous tone. Sometimes middle ear myoclonus is described as a sound slowly escalating over several minutes, only to stop abruptly. This cycle may repeat and these slowly waxing and abruptly stopping episodes may occur frequently throughout the day. The etiology of this condition is attributed to the myoclonic contraction of one of the two middle ear muscles, namely the stapedius and/or tensor tympani muscles [1–3]. Given the relative rarity of the condition, the majority of published literature on this condition takes the form of case reports or small case series. In 2013, Park et al. [4] reported the largest known series with 58 patients treated for middle ear myoclonus.

Conservative and medical therapy is thought to be first line of treatment utilizing muscle relaxants, anticonvulsants, zygomatic pressure maneuvers, simply reassurance, or even botulinum toxin [1, 4–7]. Surgical transection of the stapedius and/or tensor tympani tendons is an excellent therapeutic option in patients who fail conservative therapy, have breakthrough symptoms, or simply desire a more permanent solution [1, 3–5]. The success rates of surgical tenotomy are very high but not universal. Many authors report nonselective transection of both tendons [3–5, 8–10]. Others report selective section of only one of the two tendons based on additional clinical signs and symptoms or observations made intraoperatively with the patient awake [2, 11, 12]. A body of literature exists on how to recognize pure stapedius myoclonus or the* tensor tympani syndrome* [13]; however, the distinction is often difficult to make clinically, leading to the frequent decision to perform tenotomy of both tendons in one sitting. Stapedius and tensor tympani tenotomy has also been described in the treatment of Meniere's disease, although these causal relationships are not clearly understood [14–16]. Other reported indications for tensor tympani tenotomy include access to the anterior epitympanic recess and releasing a medialized malleus in tympanoplasty and ossiculoplasty [16].

The traditional transcanal microscopic approach to stapedius tenotomy involves raising a tympanomeatal flap, curetting the scutum for exposure in most cases, followed by transection of the stapedius tendon. Visualization of the tensor tympani tendon however is more challenging due to its relatively less accessible location. While the microscope provides superb visualization and magnification, it is a line-of-sight instrument, and many of the recessed areas of the tympanic cavity are not accessible to inspection. Different techniques are reported for adequate exposure and transection of the tensor tympani tendon using the transcanal microscopic approach [1, 2]. Blindly sliding a knife between the long processes of the incus and malleus handle to cut the tendon after raising a traditional flap is one approach that can be suboptimal as the entire tendon may not be in view [1]. Alternative approaches include extending the tympanomeatal flap superiorly and anteriorly with elevation of the drum off the malleus handle for better exposure [1] or approaching the tendon after raising an anterior tympanomeatal flap [2] which is not advisable. Using endoscopic ear surgery techniques, the tensor tympani tendon can be viewed clearly in its entirety without the need for excessive dissection.

Endoscopic ear surgery is gaining more momentum and popularity in the community of otologic surgeons and has contributed to the evolution of minimally invasive otologic surgery. The wide angle of view provided by the rigid Hopkins rod endoscopes allows the surgeon to readily visualize the middle ear anatomy, including some of the hidden recesses, and thus avoid excessive tissue dissection often done simply for exposure [17].

We designed a cadaveric temporal bone study to assess the feasibility of a completely endoscopic approach to the stapedius and tensor tympani tendons. We summarize our findings and outline a protocol for this procedure with technical tips and pearls and modifications of the procedure for those who wish to utilize the operating microscope for the elevation of the tympanomeatal flap, making the procedure essentially an endoscope-assisted procedure.

## 2. Materials and Methods

**Figure 1:**
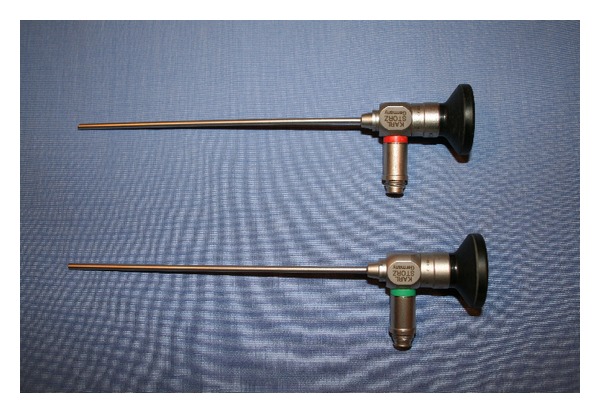
For endoscopic section of the stapedius and tensor tympani, we used the Hopkins rod rigid endoscopes, 2.7 mm diameter, zero and 30° angled, length 14 cm. These endoscopes are commonly used in pediatric endoscopic sinus surgery.

**Figure 2:**
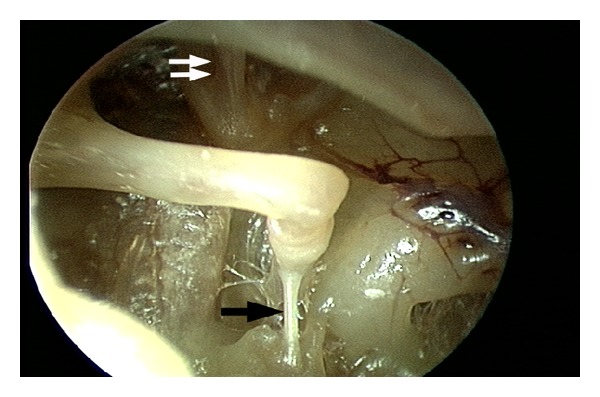
Wide view of the middle ear cavity with clearly visible stapedius and tensor tympani tendons, using a 4.0 mm, 30° rigid endoscope. The tympanomeatal flap is elevated to the level of the malleus but is not separated from the malleus. Single black arrow: stapedius tendon; double white arrow: tensor tympani tendon arises from the cochleariform process and attaches to the underside of the malleus neck.

Endoscopic section of the stapedius and tensor tympani was performed on a total of 10 temporal bones, 5 right ears, and 5 left ears. Seven of the temporal bone specimens had previously undergone canal-wall-up (intact canal wall) mastoidectomy as part of a temporal bone anatomy course. No prior middle ear work was done on any of the specimens. The temporal bones were mounted in a standard cup-shaped temporal bone holder and secured. External auditory canal debris was removed. An ear speculum was not necessary. For visualization, we used rigid Hopkins rod endoscopes, 2.7 mm 0° and 30° angled, connected to a high definition 3-CCD (3-chip) camera (Figure 1). We also tried the 4.0 mm and 1.9 mm diameter endoscopes. The image was displayed on a video screen. The endoscope is held in the surgeon's nondominant hand and the dissection is done in a one-handed fashion with the dominant hand, a technique similar to endoscopic sinus surgery [18]. Under visualization with a 2.7 mm zero degree rigid endoscope, a stapes-style canal incision was made and the tympanomeatal flap elevated using a Rosen knife. Once the middle ear space was entered, the annulus elevator was used to complete flap elevation anteriorly to the level of the malleus handle. Care was taken to preserve the chorda tympani which can sometimes be injured during flap elevation. The stapedius and tensor tympani tendons were easily and clearly identified (Figure 2). No further scutum curetting or any other dissection was needed in any of the 10 specimens. The tendons were transected using various instruments. Experience with each temporal bone was documented and summarized below (Tables 1 and 2).

## 3. Results

In one of the 10 temporal bones in which the facial recess had previously been drilled out, the stapedius tendon was absent. The tensor tympani tendon was present in all 10 specimens.

**Figure 3:**
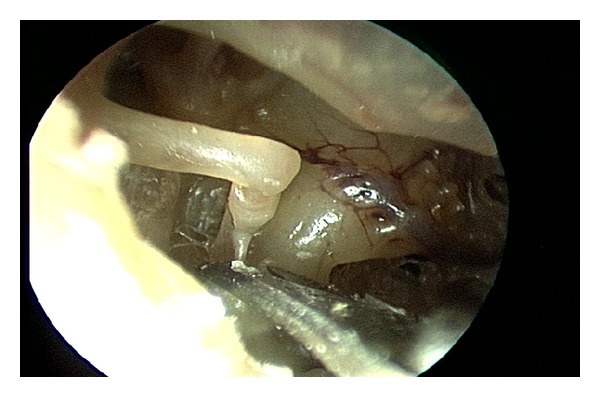
The best instrument to sever the stapedius tendon is a* straight sharp* instrument such as a Bellucci microscissors.

**Figure 4:**
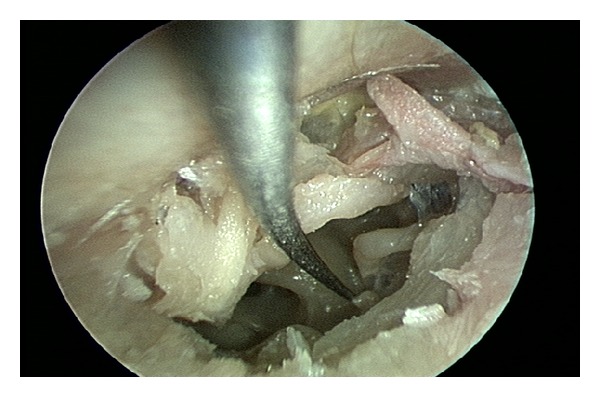
The cut ends of the stapedius tendon have memory. Here, the cut ends are deflected away from each other with a pick to create a gap and prevent reanastomosis which could potentially lead to recurrence of symptoms.

The stapedius tendon was easily visualized with a 2.7 mm 0° endoscope and in contrast to microscopic procedures, curetting of the scutum was never needed for exposure. The stapedius tendon was cut using several different sharp and blunt, straight and angled instruments (see Table 1). The stapedius tendon was sharply transected using Bellucci microscissors in 6 cases, an angled joint knife in 2 cases, and a blunt gently curved pick in 2 cases. Blunt tenotomy with a curved pick required greater force than the sharp techniques and resulted in incudostapedial joint separation in one case. The best instrument to sever the stapedius tendon appears to be a* straight sharp* instrument such as a small Bellucci scissors (Figure 3). After the stapedius tenotomy, the two ends appear to have significant memory and tend to realign. This may potentially result in healing and recurrence of pulsatile tinnitus. Using a Wullstein pick, the cut ends of the stapedius tendons were deflected from one another to create a gap (Figure 4).

**Figure 5:**
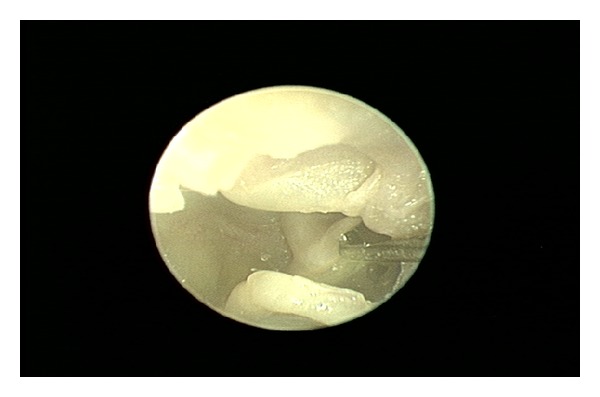
In this specimen with a narrow external auditory canal and significant anterior bony bulge, a smaller, 1.9 mm diameter, 30° endoscope was used to raise the tympanomeatal flap and visualize the tensor tympani tendon.

**Figure 6:**
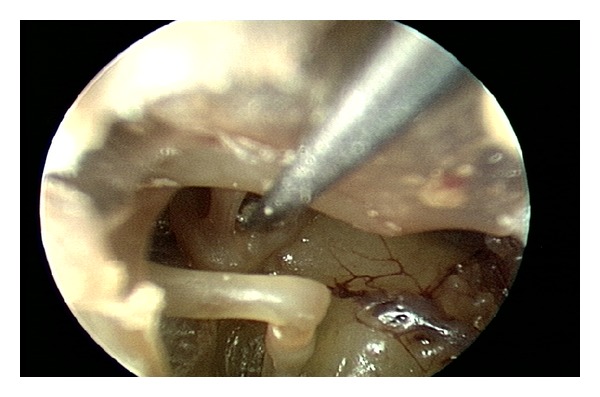
The best instrument to sever the tensor tympani tendon is a* curved sharp* instrument such as a joint knife used in stapedotomy.

The tensor tympani tendon was adequately visualized with a 2.7 mm 0° endoscope in 8 of the 10 bones, and with a 30° endoscope in all 10 cases. In specimen number 5, the space between the long process of the incus and the malleus handle was narrow and the zero degree scope did not have the proper angle to visualize the tendon. In specimen number 10, the external auditory canal was quite narrow with a protruding anterior bony bulge. The 2.7 mm 30° endoscope could not be maneuvered into this narrow canal, however a 1.9 mm 30° scope was successfully used to raise the tympanomeatal flap and fully visualize the tensor tympani (Figure 5). The tensor tympani tenotomy was performed using various sharp and blunt instruments. The tensor tympani was cut with a sharp curved joint knife in 8 cases and Bellucci microscissors in one case. The Wullstein needle was used for a blunt tenotomy in one case, which required excessive force and led to instability of the incudomalleal joint (see Table 2). The best instrument to sever the tensor tympani tendon appears to be a* curved sharp* instrument such as a joint knife used in stapedotomy (Figure 6). In all 10 cases, the Wullstein pick was used under endoscopic guidance to assure complete transection of the tendon. The two ends of the tendon were again deflected from each other using a curved pick to assure that they do not reapproximate.

## 4. Discussion

The stapedius tendon arises from the pyramidal eminence on the posterior wall of the middle ear cavity and inserts on the stapes capitulum. Its function is to alter the impedance of the ossicular chain and so contribute to the wide dynamic range of the normal ear, essentially by dampening loud sounds before the sound energy is transferred to the cochlea. The traditional transcanal microscopic approach to stapedius tenotomy involves raising a posterior tympanomeatal flap, curetting the scutum for exposure in most cases, and transection of the tendon. Our surgical protocol for endoscopic stapedius tenotomy is as follows.

- Inject the canal walls with epinephrine solution under endoscopic view with a rigid endoscope.
- Elevate a posteriorly based tympanomeatal flap. A small epinephrine-soaked cotton ball can help with hemostasis during this one-handed technique.
- Identify and preserve the chorda tympani nerve.
- Identify and cut the stapedius tendon with a* straight sharp* instrument such as a Bellucci microscissors. Curetting the scutum for exposure is not necessary with the endoscopic approach in our experience with 9 temporal bones.
- Deflect the two cut edges of the tendon with a blunt pick to avoid realignment.

For the stapedius tenotomy, the endoscopic approach offers only a small benefit over the traditional microscopic approach, specifically, there is no need to curette the scutum for exposure.

The tensor tympani courses in a semicanal paralleling the Eustachian tube, continues along the floor of the tympanic cavity, near the tympanic segment of the facial nerve, then turns 90° and emerges from the cochleariform process, attaching to the underside of the malleus neck and manubrium. Visualization of this tendon is more difficult as it is located under the malleus and the angle of view passes through the isthmus between the long process of the incus and malleus handle in the superior mesotympanum. There are several different approaches reported to transcanal microscopic tensor tympani tenotomy. Grobman et al. report raising a traditional tympanomeatal flap and sliding a cataract knife between the long processes of the malleus and incus to cut the tendon under direct vision to avoid injury to the facial nerve. Their alternative suggested approach is to extend the superior aspect of the tympanomeatal flap anteriorly, leaving the tympanomeatal flap attached to the umbo, to allow approach to the tendon anterior to the malleus [16]. In this technique, the tympanic membrane is sharply separated from the malleus, exposing the area anterior to the manubrium. Loader et al. also report raising an anterior tympanomeatal flap for exposure of the tendon and its transection [15].

We suggest the following steps for endoscopic tensor tympani section based on our experience with 10 cadaveric temporal bones.

- Under endoscopic view, inject the canal walls with an epinephrine containing solution in the standard fashion for hemostasis.
- A draped microscope should be available for surgeons who are not yet comfortable with this technique. A standard otologic microscope should be available for all but the most experienced endoscopic ear surgeons.
- Under endoscopic view, elevate a posterior stapes-style tympanomeatal flap using standard tympanoplasty instruments. Hemostasis is particularly important in this one-handed dissection technique. Experienced endoscopic ear surgeons find that using a small epinephrine-soaked cotton ball can help with hemostasis during flap elevation.
- Identify and preserve the chorda tympani nerve during flap elevation.
- The tensor tympani tendon can be identified with the 0° endoscope in most cases; however it is best to switch to a 30° scope for better view of the tendon.
- The tensor tympani is severed using a* sharp curved* instrument (e.g., joint knife) directing the sweeping motion away from the facial nerve. Several passes may be necessary as the tensor tympani tendon can be thick and fibrous in some cases.
- Inspect the cut edges to assure complete separation and deflect the cut ends with a blunt pick or other instrument to avoid realignment and potentially reanastomosis.

The benefits of the endoscopic approach to tensor tympani tenotomy over the microscopic approach are significant as surgical access to the tensor tympani is more challenging using the standard techniques. The endoscopic approach allows the surgeon to perform the tenotomy without extended flap elevation off the malleus or other excessive tissue dissection. With the availability of angled endoscopes with different shaft diameters, the tensor tympani can be visualized through the space between the long process of the incus and malleus handle, even when the gap is narrow. No blind maneuvers are necessary and the surgeon can visually confirm complete transection of the tendon and separate the two ends of the cut tendon with a gently curved pick to prevent reanastomosis. The tenotomy should be performed sharply as blunt techniques can lead to ossicular chain disruption and risk injury to adjacent structures. Deflection of both ends of the stapedius tendon is an important step as well, as the tendon has memory and tends to snap back in place.

Anatomical variants such as a narrow external auditory canal, prominent anterior canal bulge, narrow space between the long process of the incus, and malleus handle all pose challenges in performing stapedius and tensor tympani tenotomy; however with the endoscopic approach, these difficulties are surmountable and can be overcome by simply using a narrower endoscope, without having to perform any additional dissection in all but the most severe cases. We recommend using the largest diameter endoscope that can easily be passed and maneuvered in the ear canal. The endoscope tip is not passed into the middle ear space and rests near the tympanic ring. Ideally a 4.0 mm diameter rigid endoscope is used in patients with large ear canals; however in patients with a degree of congenital ear canal stenosis, prominent anterior bulge, exostoses, scarring, or limited ear canal access for other reasons, a smaller diameter rigid endoscope can be used. We found that the 2.7 mm diameter, 14 cm long endoscope worked great in most cases with excellent optics when used with a high-definition, 3-CCD (3-chip) camera. The 1.9 mm diameter endoscope was also used successfully to complete the entire operation in one case, though a clear decline in optics quality was noted compared to the 2.7 and 4.0 mm diameter scopes. Proper injection of the canal wall is critical in providing a dry field, since in endoscopic ear surgery, the surgeon cannot use the suction and a dissecting instrument at the same time. This is similar to endoscopic sinus surgery.

This study has demonstrated proof-of-concept and feasibility of completely endoscopic stapedius and tensor tympani tenotomy with multiple benefits over the traditional approach using the operating microscope. With the endoscopic approach, curetting of the scutum is not necessary for exposure. Any extended elevation of the tympanomeatal flap off the malleus is not necessary. Transection of both tendons can be done under direct vision, rather than blindly, making sure the transection is complete and thus avoiding residual symptoms or recurrence. The chorda tympani can be easily identified and protected. If the surgeon is inexperienced with endoscopic ear surgery, the technique can be combined with a classic microscopic approach. The canal injections and elevation of the tympanomeatal flap can be performed using the standard otologic microscope. The endoscope can then be used to identify the tendons and complete the tenotomy, making it effectively an endoscope-assisted procedure. The entire procedure can be done transcanal, without the need for a postauricular approach.

## 5. Conclusion

Endoscopic stapedius and tensor tympani section is a new, minimally invasive treatment option for middle ear myoclonus that should be considered as the first line surgical approach in patients who fail medical therapy. The use of an endoscopic approach allows for easier access and vastly superior visualization of the relevant anatomy, which in turn allows the surgeon to minimize tissue dissection and avoid a postauricular approach in accordance with the principles of* functional endoscopic ear surgery* [17]. The entire operation, including raising the tympanomeatal flap and tendon section can be safely completed under visualization with a 1.9–4.0 mm diameter, zero or 30° angled rigid endoscope.

## Figures and Tables

**Table 1:** Results of temporal bone endoscopic stapedius transection

Temporal bone number	Previous mastoidectomy	Stapedius tendon visible with 0° scope	Stapedius tendon visible with 30° scope	Scutum curetting or canal drilling necessary	Comments
(1) Left	Yes	Yes	Yes	No	

(2) Right	Yes	Yes	Yes	No	

(3) Left	Yes	Yes	Yes	No	Stapedius tendon was transected bluntly with a Wullstein needle. This resulted in unintended incudostapedial joint separation A prominent anterior canal bulge made the scope insertion and endoscopic guided insertion of instruments slightly more challenging but procedure was still completed using a 2.7 mm diameter scope

(4) Left	Yes	Yes	Yes	No	

(5) Right	Yes	Yes	Yes	No	

(6) Right	Yes	No	No	No	The stapedius tendon was absent because the facial recess had been previously drilled

(7) Right	Yes	Yes	Yes	No	

(8) Left	No	Yes	Yes	No	

(9) Right	No	Yes	Yes	No	

(10) Left	No	Yes	Yes	No	A prominent anterior canal bulge and narrow ear canal made the scope insertion and endoscopic guided insertion of instruments challenging Procedure was completed using a 1.9 mm diameter scope

**Table 2:** Results of temporal bone endoscopic tensor tympani transection

Temporal bone number	Previous mastoidectomy	Tensor tympani visible with 0° scope	Tensor tympani visible with 30° scope	Extended elevation of the tympanomeatal flap needed	Comments
(1) Left	Yes	Yes	Yes	No	

(2) Right	Yes	Yes	Yes	No	

(3) Left	Yes	Yes	Yes	No	Tensor tympani was transected bluntly with a Wullstein needle. This resulted in inadvertent incudomalleal joint instability A prominent anterior canal bulge made the scope insertion and endoscopic guided insertion of instruments somewhat challenging but the procedure was still completed with a 2.7 mm diameter scope

(4) Left	Yes	Yes	Yes	No	

(5) Right	Yes	No	Yes	No	The tendon could not be seen with the 0° scope because of the narrow space between the long process of the incus and malleus handle. Better visualization was achieved with the 30° scope

(6) Right	Yes	Yes	Yes	No	

(7) Right	Yes	Yes	Yes	No	

(8) Left	No	Yes	Yes	No	

(9) Right	No	Yes	Yes	No	

(10) Left	No	No	Yes	No	The narrow ear canal required use of a 1.9 mm 30° scope rather than a 2.7 mm scope
